# Synthesis and Application of a Cationic Polyamine as Yankee Dryer Coating Agent for the Tissue Paper-Making Process

**DOI:** 10.3390/polym12010173

**Published:** 2020-01-09

**Authors:** Cesar Valencia, Yamid Valencia, Carlos David Grande Tovar

**Affiliations:** 1 Área de Investigación, Desarrollo e Innovación, Disproquin S.A.S., Calle 93 Número 7u-2a, Vía Cali-Juanchito 760021, Colombia; analista.id2@disproquin.com.co (C.V.); analista.id@disproquin.com.co (Y.V.); 2 Grupo de Investigación de Fotoquímica y Fotobiología, Universidad del Atlántico, Carrera 30 Número 8-49, Puerto Colombia 081008, Colombia

**Keywords:** coating agent, paper creping, polyamine-epichlorohydrin resin, release agent, yankee dryer

## Abstract

Tissue paper is of high importance worldwide and, continuously, research is focused on improvements of the softening and durability properties of the paper which depend specifically on the production process. Polyamide-amine-epichlorohydrin (PAE) resins along with release agents are widely used to adhere the paper to the yankee dryer (creping cylinder) in paper manufacture. Nevertheless, these resins are highly cationic and they normally adhere in excess to the paper which negatively affects the creping process and the quality of the paper. For this reason, a low cationic polyamine-epichlorohydrin coating (Polycoat 38^®^) was synthesized from a diamine supplied by Disproquin S.A.S. and epichlorohydrin. The analysis of the synthesized polymer was carried out by Fourier transform infrared spectroscopy (FTIR) and nuclear magnetic resonance (^1^H-NMR). The molecular weight of the polymer was obtained by gel permeation chromatography (GPC), physical-chemical properties such as kinematic viscosity, percentage of solids, density, charge density were measured and compared with a commercial PAE resin (Dispro620^®^) Thermal stability of the Polycoat 38^®^ and glass transition temperature in presence of a release agent (Disprosol 17^®^) were also evaluated by thermogravimetric analysis (TGA) and differential scanning calorimetry (DSC), respectively. Finally, a peel adhesion test and an absorption durability assessment were carried out together with the evaluation of the creeping efficiency of the paper by caliber and tensile measurements in a tissue (towel paper) production plant, demonstrating a superior performance in the paper creping process as compared to some commercially available products.

## 1. Introduction

**Figure 1:**
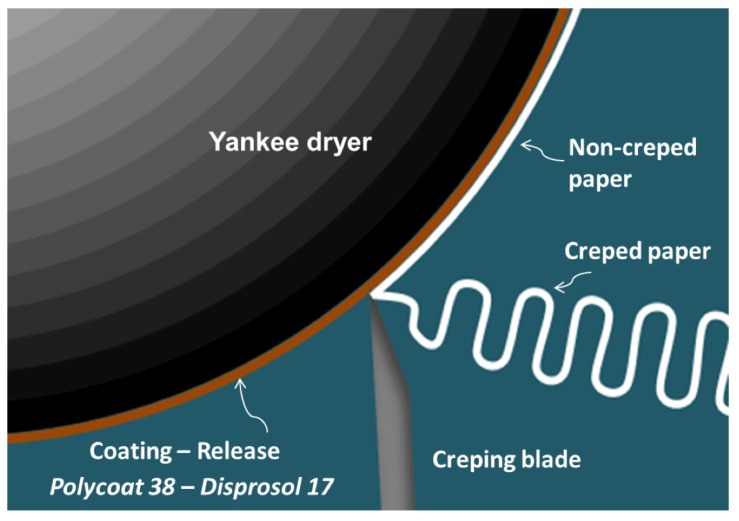
Scheme of the creping process using a coating-release agent in the yankee dryer.

Coatings are adhesive compounds which facilitate the operation of the creping machines since they allow the paper to adhere to the yankee dryer’s surface at the last step of the tissue paper conversion [1]. These are used in conjunction with a release agent that controls the hardness of the coating and its adhesion, allowing the demolding of the paper once in contact with the creping blade as shown in Figure 1 [1].

Coating agents commercially available, such as Discrepel HRC^®^, Kymene 557H^®^, Rezosol 8223^®^, and others [2], are synthesized from various available additives, such as polyamine-amide resins mixed with polyvinyl alcohols and ethoxylated alcohols [2]. Unfortunately, they tend to develop less re-wettable hard coatings due to the high cationic charge of the polymer [3,4]. The described situation causes a strong adhesion and blade vibrations, which in turn generates non-uniform creping and profile of the paper, blade wear, and damage to the surface of the drying cylinder [5]. On the other hand, the most common release agents usually have the combination of fatty alcohols, glycerol, and non-ionic emulsifying agents such as lecithin [6,7].

The main limitation is that all the components must be completely emulsified along with the coating in the yankee’s surface during application since the hardness and adhesiveness should be equilibrated depending on the needs [7,8]. The previous requirement implies that during the application the emulsions must be stable and homogeneous, which normally does not occur when the most common release agents are used [8]. In general, these types of emulsions are easily separated and their application in the yankee leads, for example, to non-homogeneous coating, which is traumatic for the drying process affecting the formation of the tissue paper rolls and also yankee’s surface could be deteriorated [5,7]. The shelf-life of the doctor creping blade could be reduced and the different adherence intensity to the paper sheet deteriorates its creping process and the final quality of the paper [5,7].

Therefore, there is a great demand for a crepe coating-release couple that remains soft and re-wettable under the drying conditions found in creping [1,7]. It is necessary that produces high creping efficiency while generating a high-quality paper [9], less creping blade wear, and greater protection for the yankee to generate a uniform release of the paper and that does not produce bursting problems on paper rolls [7].

Promising coatings that seems to accomplish these requirements are the polyamine-based resins. These resins show charge density and structures related to the fibers of the pulp which generates lower dry strength in the paper than the classical PAE [10,11]. In addition, they have less adhesion and hardness properties, controllable during the polymerization process [12] in order to have properties required by the yankee [5]. At the same time, release agents based on vegetal oil surfactants present stable emulsions that control this type of coatings once they are applied to the yankee [13,14], which inspire us for the present development.

For this reason, a new coating (Polycoat 38^®^) was designed based on a diamine and epichlorohydrin [15] to generate a cationic polyamine whose structure, viscosity, percentage of solids, charge density, and tensile measures indicates a greater adhesion-release balance. Therefore, greater rewetting properties together with the interaction of a mineral oil-based release agent (Disprosol 17^®^), allows a more stable coating on the yankee [1], with high resistance, greater smoothness, and a better absorbency of the paper while the creping took place at a paper mill of a tissue production plant [5,7,8].

## 2. Materials and Methods

### 2.1. Materials

All the reagents used in this research (U.S.P grade) were obtained from Sigma-Aldrich (Palo Alto, CA, USA) and used without further purification unless otherwise stated.

### 2.2. Synthesis of the Polyamine Resin Polycoat 38^®^

Polycoat 38^®^, which is a polyamine obtained by the A. Allen modified method [15], uses a difunctional polyamine or diamine with epichlorohydrin for the polymer synthesis with the addition of polyvinyl alcohol between 25–60 °C, and it is advantageous providing strong adhesion of the cellulosic fiber web to the dryer surface during the creping process to attain a soft, bulky tissue paper web [15,16].

The diamine was placed in a 100 kg industrial pilot reactor with double jacket coupled to a steam and a water recirculatory ice bank (Disproquin S.A.S., Candelaria, Colombia). Aqueduct water was added to the pilot in order to reach 38 wt % solids. It was then heated to 50 °C and stirred. A sample of 8.50 kg of epichlorohydrin were slowly added at room temperature with agitation. The exothermic reaction reached a temperature of 150 °C and it was stirred for 4 h. Finally, when a kinematic viscosity between 400 and 500 cP was reached, a solution of 0.1% sulfuric acid was added to stop the reaction. The resultant polyamine cationic product was stored in plastic drums [4,15,16,17,18].

### 2.3. Characterization

#### 2.3.1. Characterization of the Polycoat 38^®^

FTIR measurements were performed on an infrared equipment, Thermo brand model NIcolet 6700 (ThermoFisher Scientific, Waltham, MA, USA), using KBr tablets. ^1^H-NMR measurements were performed on a 400 MHz NMR Bruker Ultra Shield (Bruker Corporation, Billerica, MA, USA) using D_2_O as a solvent. GPC measurements for the molecular weight determination of the polymer were performed using a gel permeation chromatograph Agilent 1200 (Agilent Technologies, Santa Clara, CA, USA) with 2 intercrossed polymer columns Shodex ohpak (Showa Denko, Tokyo, Japan) as the stationary phase and NaNO_3_ 0.15 M /HCOOH 0.5 M as the mobile phase, using pullulan standards for the calibration curve and a refractive index detector. Thermogravimetric analysis was performed on a TGA-2050 thermogravimetric analyzer (TA instrument, New Castle, DE, USA) adjusted in a working temperature range between 25–400 °C. DSC measurements were made in a DSCQ 100 (TA instrument, New Castle, DE, USA). The ion demand of the resin was measured with an automatic particle charge analyzer (AFG Analytic GMBH, Leipzig, Germany). The non-volatile percentage was calculated using Equation (1).

%Solids = *W*_f_/*W*_i_ (1)

where *W*_i_ was the initial weight of the sample and *W*_f_ was the final weight after heating at 105 °C during one hour in an oven (Memmert Gmbh + Co. KG, Buchenbach, Germany).

The kinematic viscosity was measured to 500 g of the sample, using the needle 1 at 50 RPM and the respective Brookfield RVT viscometer (Brookfield Engineering Laboratories Inc., Middleborough, MA, USA). The density was calculated by weighing 1.000 mL of the sample, added with a micropipette (Eppendorf, Hamburg, Germany). The pH was measured with a Hanna pH meter (Hanna Instruments Inc., Woonsocket, RI, USA). Peel adhesion test of the coating was carried out with a horizontal tensile tester ZB-WL30 (Hangzhou Zhibang Automation Technology Co., Ltd., Zhejiang, China) to tissue hand-sheets with 30 g/m^2^ grammage, 5.5 cm long and 1.5 cm with, attached to a standardized aluminum panel 9.5 cm long and 1.5 cm width. In the procedure, 0.04 mL of the coating is added to the panel and spread with a bronze applicator. The paper is adhered to the panel at 105 °C in an oven (Memmert Gmbh + Co. KG, Buchenbach, Germany) and its exposed edge is tightened with the tensile clamp, which removes the strip at a speed of 10 mm/min and records the force required to detach it [2]. Finally, the coating durability was evaluated by the degree of swelling and erosion [5], where the coating was immersed in distilled water at room temperature. After 24 h, the coating was filtered, the water residues were dried with filter paper and wet weighted, the sample was put in an oven (Memmert Gmbh + Co. KG, Buchenbach, Germany) for 24 h at 37 °C. Its dry weight was recorded and the following Equation (2) was used for the calculation.

%Erosion = ((*W*_i_ − *W*_f_)/(*W*_i_)) × 100 (2)

where *W*_i_ is the wet weight and *W*_f_ is the final dry weight.

#### 2.3.2. Characterization of the Tissue Creping Paper

The hand sheets for the analysis of the paper were obtained folding 16 times a 2.65 m^2^ sheet of a paper roll which is then cut using an Alfa pneumatic precision cutter (Thwing-Albert Instruments Company, West Berlin, NJ, USA). As a result, 16 sheets whit 22.5 cm^2^ area and grammage between 14.5 and 15.5 g/m^2^ were obtained. Caliber of the 16 sheets was measured with a digital micrometer (testing machines Inc., Las Vegas, NV, USA). Tensile strength and elongation of 8 sheets in machine direction (MD) and 8 in cross machine direction (CD) were measured using a QC-1000 Tensile Tester (Thwing-Albert Instruments Company, West Berlin, NJ, USA).

### 2.4. Application of the Polycoat 38^®^ and the Disprosol 17^®^ in the Yankee Dryer

Both products were added trough a shower at 35 psi using two dosing pumps, the Polycoat 38 and the Disprosol 17^®^ where dosed with a 1.4:2 ratio to a 2.65 m wide and 5.2 m diameter yankee dryer working at a speed of 400 m/min, with internal steam pressure of 112 psi and an external temperature of 105 °C, where the doctor creping blade exerts a pressure of 45 psi on the product which allows 14.5–15.5 g/m^2^ grammage paper rolls with weights between 950–1150 kg to be produced at a speed of 336 m/min, leading to the fabrication of triple sheet towels in a Colombian toilet paper manufacturing company. Creping and cleaning blades wear were measured using a digital microscope camera (Microview Science and Technology Co., Ltd., Beijing, China).

**Figure 2:**
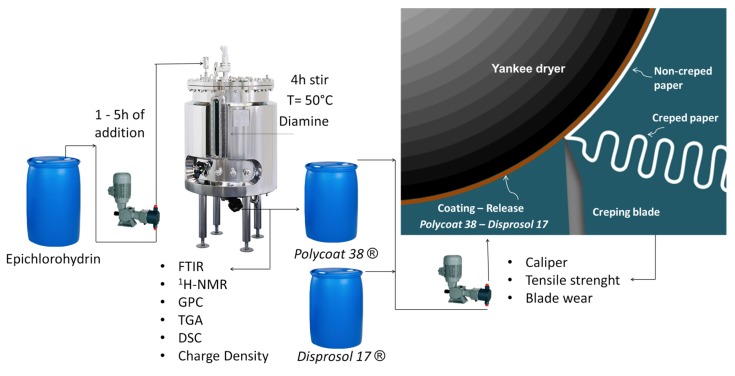
Graphical abstract of the synthesis and application of the *Polycoat 38*^®^ coating in the yankee dryer.

To complement the previous information, a graphical abstract of the methodology used from the coating synthesis to its application in the paper mill can be observed in the Figure 2.

## 3. Results and Discussion

### 3.1. FTIR Analysis of the Polycoat 38^®^ Resin

**Figure 3:**
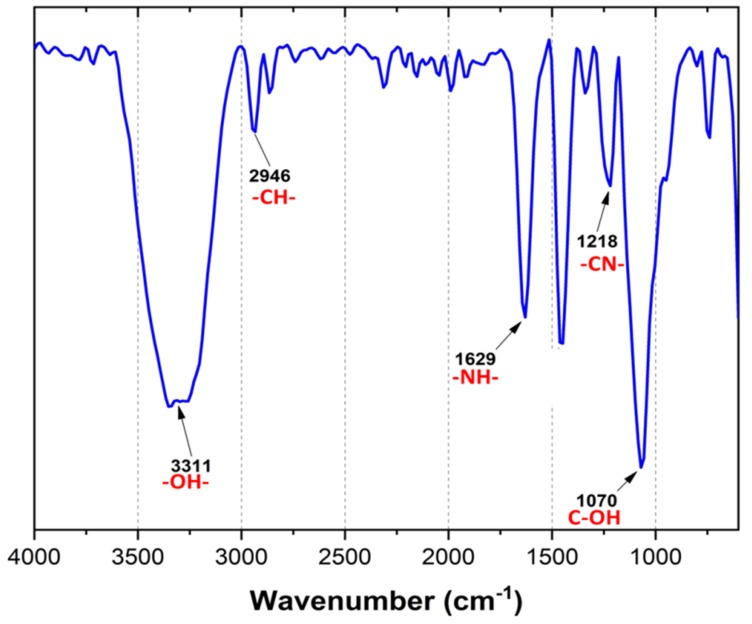
Fourier transform infrared spectroscopy (FTIR) spectrum of the polyamine (*Polycoat 38*^®^) resin synthesized.

The FTIR spectrum of the polymer resin is presented in Figure 3. The characteristic bands of the cationic polyamine [10,19,20] are observed. At 3311 cm^−1^ the broad tension vibration band of the O–H bond is observed. The band at 1070 cm^−1^ corresponds to the C–O bond in the C–OH group that allows the interaction with the fiber and that is part of the azetidine ring [21]. On the other hand, at 1218 cm^−1^ is the tension band of the C–N bond presented in the quaternary nitrogen of the azetidine ring [10], also, the vibration band of the N–H caused by the interaction between the added acid and the groups of free amines appears in 1623 cm^−1^ [15] Finally, at 2946 cm^−1^, the C–H tension vibration band of the methylene groups in the main chain of the polymer is observed [22].

### 3.2. ^1^H-NMR Analysis of the Polycoat 38^®^ Resin

**Figure 4:**
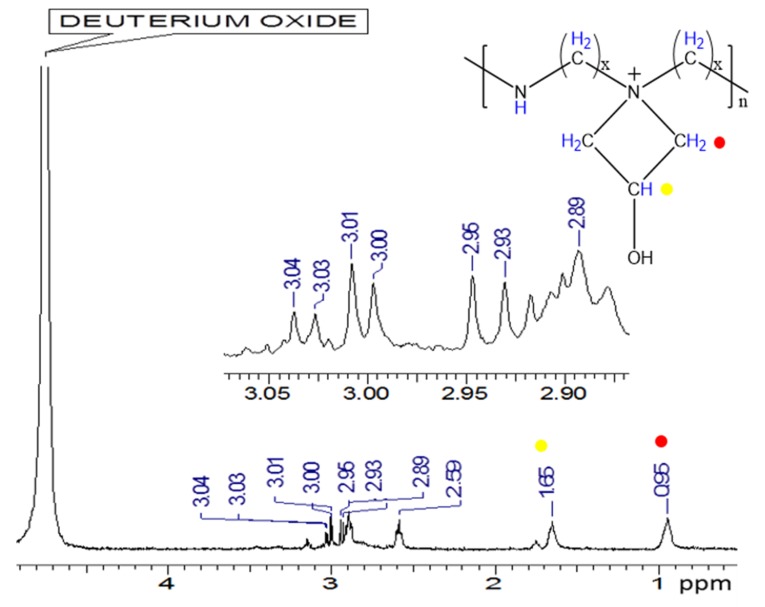
Nuclear magnetic resonance (^1^H-NMR) spectrum of the polyamine (Polycoat 38^®^) resin synthesized.

In the ^1^H-NMR spectrum (Figure 4), the characteristic signals of the protons presented in the coating resin can be observed [23–25]. The signals at chemical shifts of 0.96 and 1.66 ppm correspond to the protons of the CH_2_ and CH groups of the azetidine ring, where quaternary nitrogen are generated and relate with OH groups at the end of the ring [25]. These OH groups tend to form hydrogen bonds with the cellulose and hemicellulose (polyoses) allowing the adhesion of the paper to the yankee and a posterior detachment once it comes into contact with the creping blade [24]. On the other hand, the multiplet presented between 2.89 and 3.04 ppm are characteristic of the protons presented in the CH groups of the main polymer backbone [4,25].

Finally, a signal from the NH and the OH protons of the chain would be expected around 8 ppm. However, the signal is not observed due to the presence of the deuterated solvent that causes an exchange of protons with the hydrogens of this groups and decreases the intensity of the signals [25]. The figure also shows the possible structure of the cationic polymer and its corresponding azetidine ring [4].

### 3.3. Molecular Weight Determination of the Polycoat 38^®^ Resin

The molecular weight of the resin was calculated using gel permeation chromatography technique. Retention time of the peak corresponding to the polyamine yield an average molecular weight (Mw) of 3131 g/mol and an average number molecular weight (Mn) of 2890 g/mol.

Mw was higher since it corresponds to higher molecular weight chains, while Mn is sensitive to low molecular weight chains [22]. On the other hand, these values allowed to elucidate the polydispersity index (PI), which in this case corresponded to 1.08. This value is close to the unit and indicates that the lengths of the chains are practically equal [26]. That will produce a homogeneous distribution on the surface of the yankee, which in turn will generate an optimal contact with the sheet of paper to crepe without the presence of holes caused by spaces between chains of high and low molecular weight [27]. Likewise, the distribution of the cations across the polymer are uniform decreasing the residual epichlorohydrin of the synthetized resin which is an environmental requirement for these processes [28].

### 3.4. Thermogravimetric Analysis (TGA) of the Polycoat 38^®^ Resin

**Figure 5:**
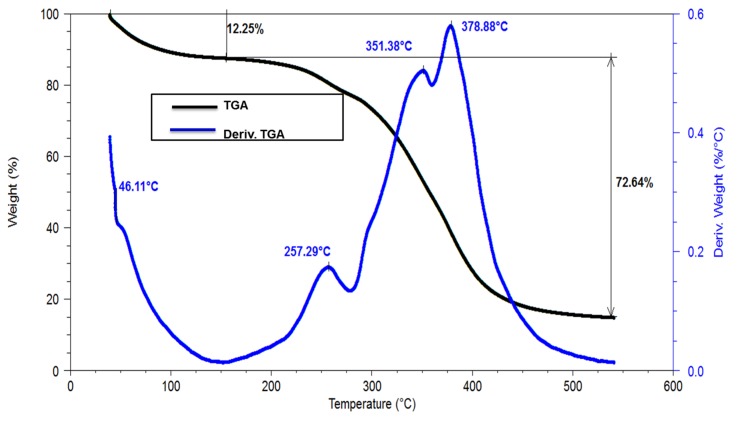
Thermogravimetric analysis of the polyamine (*Polycoat 38*^®^) resin synthesized.

Figure 5 shows the weight loss of the polymer in function of the temperature. The first loss is due to the amount of water retained in the interstices of the polymer which tend to evaporate between 45 and 100 °C [29]. The second loss is observed between 351 and 378 °C, corresponding to the rupture of the main chains in the polymer, leading to its degradation [25]. The given temperature is higher than the working temperature of the surface of the yankee [5], which indicates that there will be no problems due to the deterioration and subsequent breakage of the main chains of the polymer and the opening of the azetidine rings [5], needed to adhere the paper through OH cellulose groups on the yankee’s surface [30].This indicates that a loss of durability of the coating will not be present since the yankee surface temperature is lesser than the coating degradation temperature [5], meaning that the coating performance will not be affected, so loss of bulk in the finished paper rolls and cross machine direction profile issues of uniformity in the produced paper roll will not be observed [7].

### 3.5. Differential Scanning Calorimetry (DSC) of the PAE Resin

The differential scanning calorimetry analysis is of utmost importance because it shows the vitreous transition temperatures (*T*_g_) of the resin [31]. The value of *T*_g_ on the central axis is between the range in which the behavior of the polymer is between glassy and rubbery [31]. The *T*_g_ needs to be greater than the yankee working temperature, otherwise the coating will not be soft enough to prevent blade chattering [5,7], wearing it out and producing a direct contact with the yankee by loss of thickness of the coating layer and a “smooth” crepe with more space between the crepe pockets [5].

**Figure 6:**
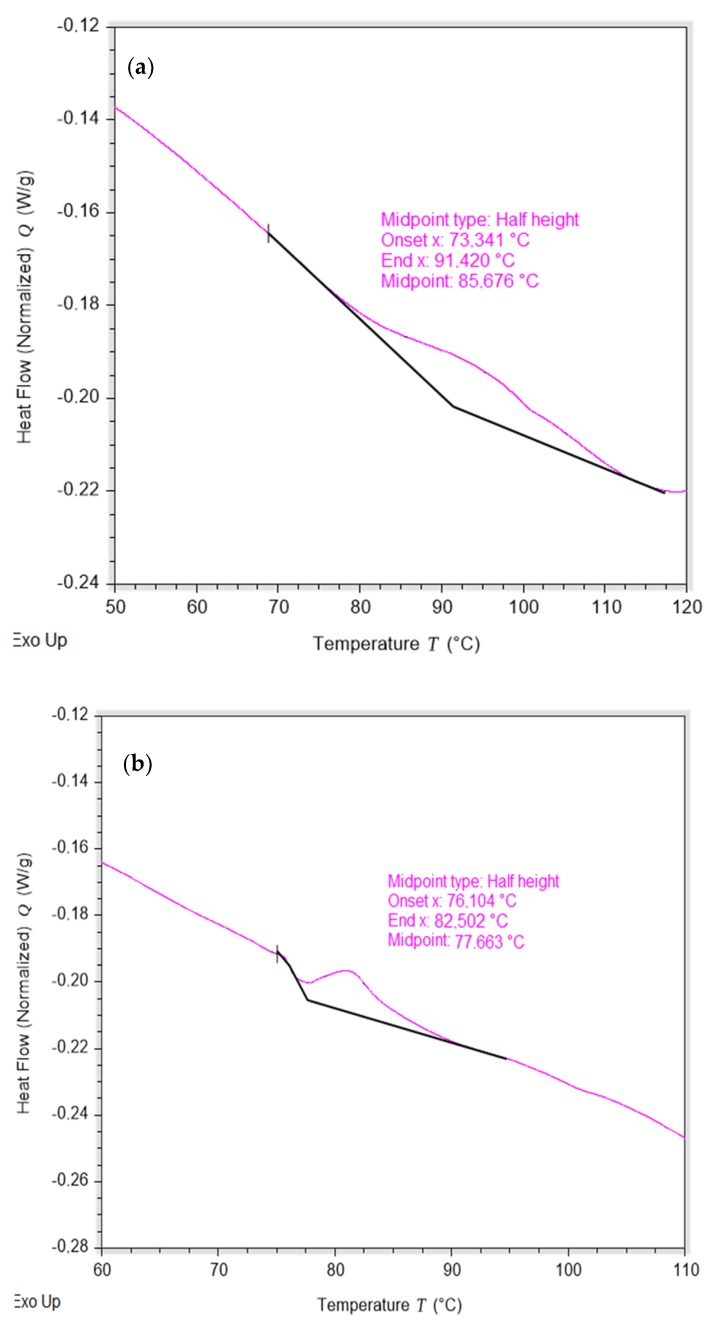
Differential scanning calorimetry (DSC) analysis of (**a**) the *polycoat 38*^®^ resin synthetized and (**b**) emulsion of *polycoat 38*^®^ and *Disprosol 17^®^.*

In this case, the temperature of the yankee where the product was proven exceeded 100 °C and it can be seen in Figure 6 that the *T*_g_ of the coating alone and emulsified with release in a proportion similar to that used in the yankee were 85.7 and 77.7 °C, in which the polymer behaves like a thermoplastic rubber and is soft enough to produce high quality paper [31]. This DSC behavior was similar to other polyamide-based resins as reported by Huang K. et al. [32].

It should be noted that each yankee operates at different temperatures depending on the requirements of the paper mill. If a slightly lower temperature is required, then the coating *T*_g_ should be slightly reduced by the addition of the release in a controlled way. For this reason, it is preferable that the temperature range of the product is not so wide in order to easily achieve subtle changes in the *T*_g_ of the polymer when it is required [31,33].

This behavior is observed in Figure 6 where the addition of *Disprosol 17*^®^ to the coating decreases the *T*_g_ values slightly by increasing the softening of the chemical duple which was evidenced on the brittle property of the polymer under this temperature, generating a high adhesion coating that can be easily coupled to yankee dryers working at lower temperatures [33].

### 3.6. Relative Adhesion of Polycoat 38^®^

The relative adhesion of the coating was measured by the peel adhesive method [2,5] and although the peel produced is not similar to that of a crepe blade, it is possible to measure the force required to release the paper without breaking it [2]. In the procedure, the metallic surface of the yankee is simulated at 105 °C by a metal panel at the same temperature covered with coating and attached to a low grammage tissue paper in order to compare the Polycoat 38^®^ with other commercial coatings [2,5,15].

Table 1 shows the results of relative adhesion of different coatings together with Polycoat 38^®^.

As observed in Table 1, the adhesion of the Polycoat 38^®^ is lower than a PAE such as *Dispro620*^®^ [12], a high adhesion can lead to paper tearing during production because the tensile force required to tear paper of grammage 15 g/m^2^ in general, is close to 600–1000 gF/3in, which is a value far below than the given by the PAE. Likewise, other polymer-based coatings such^®^ as Polydadmac or Discrepel HRC^®^ can be highly cationic, which generate a strong adhesion of the paper to the yankee so that during their addition, a large amount of release would have to be added and thus decrease the adhesion but increase costs [10,11].

Adding for example, Disprosol 17^®^ to the Polycoat 38^®^, we observed a decrease of 10% in the adhesion of the coating, which apparently resembles more the dry strength of low grammage paper. Thus, it was possible to balance the adhesiveness of the coating on the yankee as reflected during the application and production of the paper [7,8].

### 3.7. Durability Test of the Polycoat 38^®^

The swelling erosion test of Polycoat 38^®^ showed a polymer degradation of 95.9% after 24 h when comparing the initial swollen weight and the dry weight at 37 °C using Equation (1) [5].

To evaluate the rewetting of the polymer, the time that takes for the yankee to rotate 360° was used since it is the time in which another layer of coating is sprayed again [3]. This time value was equivalent to 0.26 s and was obtained from the data provided in Section 2.4. Using the result of the erosion of the polymer in 24 h and the yankee rotation time, a value of 0.0003% loss of durability during one yankee rotation was obtained. This result is practically negligible and means that there would not be degradation caused by humidity during the Polycoat 38^®^ uses [4,5].

### 3.8. Quality Parameters of the Polycoat 38 Resin and Disprosol 17 Release Agent

Physicochemical properties of the coating and the release agents were determined according to the quality requirements of both products and are presented in Table 2.

The parameters shown in Table 1 provide information for industrial purposes and certain parameters such as pH must be within a range that can affect the performance of the yankee.

For example, the water of the tissue paper mills has a relatively neutral pH, so the mixture of both products must be maintained between 5–9 to avoid deterioration in the yankee [34].

On the other hand, other parameters such as the viscosity of the products provide information on the product itself. A high viscosity is related to a lower flexibility of the polymer which may decrease the adhesiveness of the coating [35]. The release agent may be necessary to decrease the viscosity of the final product and its smoothness to achieve a more uniform creping [5,33].

Another important property for this type of cationic polymer-based resin is the charge density. Charge density is related to the percentage of azetidine rings formed in the resin which are those that adhere to the fiber when the paper passes through the yankee [30]. A higher charge indicates greater adhesiveness and although it is a desirable property an excess may cause an excessive paper sticking causing deterioration of the coating when comes in contact with the creping blade [7]. This undesirable property is generally provided by top-charge resins such as PAEs, which have been presenting these problems despite their broad application [3].

When comparing the charge density of a PAE such as Dispro 620^®^ manufactured at Disproquin S.A.S (2700 mEq/L) with Polycoat 38^®^ (1800 mEq/L), it was inferred that there is a bigger amount of azetidinium groups in the PAE compared to the Polycoat 38^®^. These groups are the reason of the interaction with the fiber [24] and although a good adhesion to the fiber is necessary, an excessively high charge generates a very strong adhesion, which leads to difficulties in the demolding and reduces the creping efficiency [3,4,5]. For this reason, the use of a PAE was discarded and the application was done with the Polycoat 38^®^, which generated paper with desirable properties, as explained previously at Section 3.8 [36].

### 3.9. Application of the Polycoat 38 Along with Disprosol 17 as Yankee Dryer Coating-Release Agent

The interaction of the coating-release agents with the paper and the yankee dryer leads to a complex chemistry that has not been fully developed. In addition, this interaction is affected by multiple process such as changing a felt, a blade, cleaning a mesh or modifying a pick-up pressure [13]. For this reason, measurements of the effectiveness of products at the laboratory level are only approximate and are not necessarily reflected in the application [37]. Therefore, the analysis that is performed during production goes hand in hand with the experience of the operator and the process quality analyst. Thus, the application of the product was done starting at a ratio of 1:1 until reaching a dose of 7 mL/min of Polycoat 38^®^ and 10 mL/min of Disprosol 17^®^ (1:1.4), which allowed an optimal profile of the paper roll in obtaining at a speed of 352 m/min. A balanced profile indicates that there are no bulges in the forming-roll caused by a poor distribution of chemicals or an unstable emulsion or a swelling in the paper.

**Figure 7:**
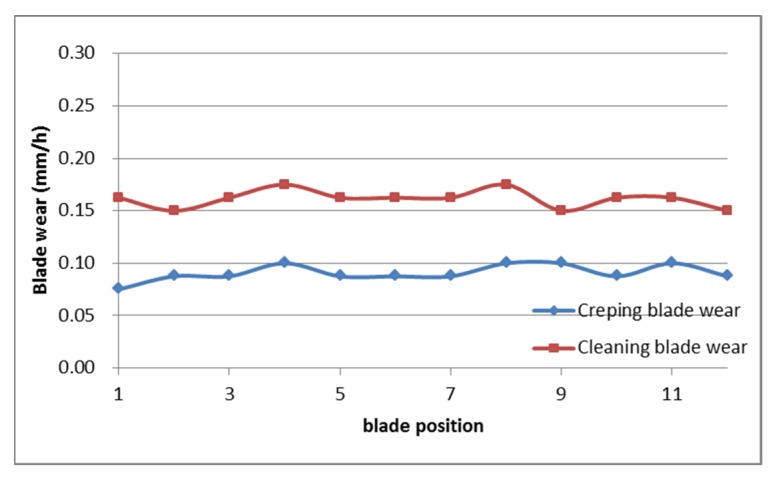
Creping and cleaning blade wear as a function of time.

On the other hand, during the application there were no bursts of paper in formation which indicates that there is an optimal adhesiveness generated by the Polycoat 38^®^ and a good relationship between this and the release capacity of Disprosol 17^®^ once the paper is creped. In addition, there was little noticeable wear on the creping and cleaning blades as shown in Figure 7.

As shown in Figure 7, the wear of both blades was minimal and even along their axes (position 1 to 11). In addition, when the creping blade was removed, did not present residue. This shows that the product covers uniformly the surface of the yankee and does not leave residue on the blade that could interfere with creping [5]. On the other hand, the cleaning blade tends to have a slightly higher damage compared to the creping blade since it removes the coating-release residue in the yankee and its pressure against the cylinder is higher.

### 3.10. Quality Parameters of the Tissue Creping Paper Obtained

#### 3.10.1. Caliber of the Tissue Creping Paper Obtained

Caliber is one of the most important properties of tissue paper as it gives a ratio of the thickness of each sheet [38]. This parameter relates to the angle of the ‘pockets’ or ‘crepes’ per unit length [5]. Thus, the paper mill must maintain the caliber of the triple sheet tissue at a value lower than 12.0 mm/100 folds otherwise the creping would be irregular and the produced paper would be rough [38]. Caliber also allows to evaluate the quality of the coating. If caliber remains high throughout the application the adhesiveness of the coating would not be optimal [15], but if it remains low and rises in a short period of time, it would indicate that the hardness of the coating wears very quickly the blade [5].

The addition of the Polycoat-Disprosol agent generated paper with a caliber of 9.4 mm/100 folds. This indicates that the adhesiveness of the Polycoat 38^®^ is of high quality and suggest that the wear of the blade was not representative and allowed to produce soft paper with optimal crepe [14].

#### 3.10.2. Tensile Strength and Stretch of the Creping Paper Obtained

The measurement of the tensile strength of the paper does not allow to measure the efficiency of the coating-release directly, but it does allow to obtain the stretch force in the direction of the machine (*MDS*) [39]. With this value is possible to obtain the relative efficiency of a coating by using Equation (3) of stretch per creping unit [5], where *CE* is the relative coating efficiency and %*C* is the creping percentage, which in turn is obtained using Equation (4) [5].

*CE* = *MDS*/%*C* (3)

%*C* = (*YS* − *RS*/*YS*) × 100% (4)

where *YS* is the speed of the yankee and *RS* is the speed of the roll or winding cylinder of paper. When replacing these variables with the speed values given in Section 2.4 a result of 16%*C* is obtained. This %*C* was used in the Equation (3) along with the *MDS* which was 22% (calculated with the tensile tester), to obtain the coating efficiency of 1.4 *MDS*/%*C*.

Nevertheless, this efficiency value does not indicate much if it is not compared with the efficiency of other coatings agents found in the literature. In this case, the efficiency of the Polycoat 38^®^ appears to be 45% higher than other coating systems such as the reported by Archer S. et al. [5] with a value of 0.94 *MDS*/%*C*, meaning that Polycoat 38^®^ have the desired behavior of adhesiveness, softness, and durability for a high-quality creped tissue [9,39,40,41].

## 4. Conclusions

A polyamine-based resin (Polycoat 38^®^*)* was synthetized by a modified A. Allen method, and was demonstrated to fulfill the requirements as a coating adhesive due to the presence of azetidine groups in its structure which allows interactions with polyoses of creped tissue paper. The presence of these groups was elucidated by FTIR, ^1^H-RMN, and charge density analysis. These analyses, together with the GPC and thermogravimetric analysis permitted to know the polymer molecular weight, structure, its thermal stability, and its *T*_g_ along with Disprosol 17^®^ (release agent) at the working temperature of a yankee dryer, were the coating-release agent was applied at a ratio of 1:1.4 respectively.

The addition of the chemical couple (coating-release) allowed a uniform profile along the paper roll of the tissue paper and minimal wear on the creping and cleaning blades evidenced in the low caliber of the paper and quality parameters. This also relates to the effectiveness of the coating which was 45% higher than others previously reported.

## Figures and Tables

**Table 1:** Relative adhesion of different coatings commercially available.

Coating	Relative Adhesion (gF/3in)
*PAE Dispro620* ^®^	2823
*Discrepel HRC* ^®^	1595
*Glyoxilated Polydadmac*	1179
*Polycoat 38* ^®^	1078
*Polycoat 38:Disprosol 17^®^ (1:1,4)*	966
*CR180* ^®^	N/A

N/A: No adhesion.

**Table 2:** Quality parameters of the Coating (Polycoat 38) and the release agent (Disprosol 17).

Parameter	Polycoat 38^®^	Disprosol 17^®^
Appearance	Liquid	Liquid
Color	Ambar	Ambar
pH	3.00–5.00	6.00–8.00
Viscosity	<600 cP	<400 cP
Total solids	38.00%	100%
Density	1.01–1.03 g/mL	0.80–0.90
Charge density	1800 mEq/L	N/A
Dispersion	N/A	100% in water

N/A: Not applied.
